# Visualization and appearance of artifacts of leadless pacemaker systems in cardiac MRI: An experimental ex vivo study

**DOI:** 10.1007/s00508-018-1334-z

**Published:** 2018-05-23

**Authors:** Christoph Edlinger, Marcel Granitz, Vera Paar, Christian Jung, Alexander Pfeil, Sarah Eder, Bernhard Wernly, Jürgen Kammler, Klaus Hergan, Uta C. Hoppe, Clemens Steinwender, Michael Lichtenauer, Alexander Kypta

**Affiliations:** 1 Clinic of Internal Medicine II, Department of Cardiology, Paracelsus Medical University of Salzburg, Salzburg, Austria; 2 Department of Radiology, Paracelsus Medical University Salzburg, Salzburg, Austria; 3 Division of Cardiology, Pulmonology, and Vascular Medicine, University Duesseldorf, Medical Faculty, Duesseldorf, Germany; 4 Clinic of Internal Medicine III, Friedrich Schiller University Jena, Jena, Germany; 5 1st Medical Department—Cardiology, GeneralHospital Linz, Johannes Kepler University School of Medicine, 4020 Linz, Austria

**Keywords:** Leadless pace maker, Micra, Cardiac MRI, Artifacts, Ex vivo model

## Abstract

**Background:**

Leadless pacemaker systems are an important upcoming device in clinical rhythmology. Currently two different products are available with the Micra system (Medtronic) being the most used in the clinical setting to date. The possibility to perform magnetic resonance imaging (MRI) is an important feature of modern pacemaker devices. Even though the Micra system is suitable for MRI, little is yet known about its impact on artifacts within the images.

**Objective:**

The aim of our ex vivo study was to perform cardiac MRI to quantify the artifacts and to evaluate if artifacts limit or inhibit the assessment of the surrounding myocardium.

**Methods:**

After ex vivo implantation of the leadless pacemaker (LP) in a porcine model, hearts were filled with saline solution and fixed on wooden sticks on a plastic container. The model was examined at 1.5 T and at 3 T using conventional sequences and T2 mapping sequences. In addition, conventional X‑rays and computed tomography (CT) scans were performed.

**Results:**

Correct implantation of the LP could be performed in all hearts. In almost all MRI sequences the right ventricle and the septal region surrounding the (LP) were altered by an artifact and therefore would sustain limited assessment; however, the rest of the myocardium remained free of artifacts and evaluable for common radiologic diagnoses. A characteristic shamrock-shaped artifact was generated which appeared to be even more intense in magnitude and brightness when using 3 T compared to 1.5 T.

**Conclusion:**

The use of the Micra system in cardiac MRI appeared to be feasible. In our opinion, it will still be possible to make important clinical cardiac MRI diagnoses (the detection of major ischemic areas or inflammatory processes) in patients using the Micra system. We suggest the use of 1.5 T as the preferred method in clinical practice.

## Introduction

The implantation of a permanent cardiac pacemaker device (PM) is currently the only effective treatment option for symptomatic bradycardia, as evidenced by the reduction of symptoms, reduction of syncope and a decrease in overall mortality. According to the 2013 European Society of Cardiology (ESC) guidelines on cardiac pacing and cardiac resynchronization therapy, a class I indication for cardiac pacing is given for patients with persistent bradycardia due to atrial fibrillation or sinus node disease.

Within 50 years of clinical use, pacemakers have shrunk remarkably in size, while their features have developed from simple basic functions to highly sophisticated medical high-tech products. Today, typical systems consist of two components: the first is a pacemaker with integrated electronics and battery, usually implanted into a subcutaneous pocket of the pectoral region. The electrical impulse is generated within the pacemaker and is transmitted to the inner heart via one or more pacemaker leads which are usually implanted through the veins into the right ventricle. Although these devices have been shown to be safe and effective, a significant number of patients encounter complications during treatment. Short-term complications include perioperative hematomas, pneumothorax/hemothorax, valve trauma or infections of the subcutaneous pocket. Long-term complications to be mentioned are lead dislodgement, lead breaking or infections such as endocarditis or septicemia. Furthermore, the surgical extraction of leads showing any kinds of functional loss or damage, are often challenging [1].

Technical progress has set the stage for a new era of cardiac pacing, which no longer depends on leads, due to permanent intracardiac placement of a new generation of leadless pacemaker (LP) devices [2].

The Micra^TM^ (Medtronic, Minneapolis, MN, USA) transcatheter pacing system (TPS) has recently been developed [3, 4]. The Micra^TM^ TPS is a 0.8 cm^3^, 2.0 g capsule, 25.9 mm in length and an outer diameter of 6.7 mm that has features of a single-chamber pacemaker system. It is implanted in the right ventricle via a steerable transfemoral catheter delivery system using a 23 French introducer [5]. Due to its implantation into the myocardial wall via the femoral vein, the main sources of potential complications (e.g., subcutaneous pockets, permanent leads) are eliminated [6, 7].

Reynolds et al. performed the first major prospective clinical trial on the Micra system and compared it with conventional systems based on historical data [4]. In this multicenter study, a total of 719 out of 725 patients (99.2%) underwent successful implantation, without any documented case of inflammatory complications. In comparison to historical data on transvenous systems, the defined safety endpoint (freedom from system-related or procedure-related major complications) and the primary efficacy end point (percentage of patients with low and stable pacing capture thresholds at 6 months) showed similar effectiveness in the Micra system [8–12]. Early performance and effectiveness has previously been shown in a porcine model [13] and recently first clinical data have been published [14–16].

In addition to the safety and efficacy shown in the clinical trial, in a limited number of clinical cases the Micra TPS has been shown to be an effective treatment option for temporary and permanent use in patients suffering from infections of conventional devices [17–19].

Pacemakers compatible with magnetic resonance imaging (MRI) have been developed and are already part of daily clinical routine. In 2017 the Heart Rhythm Society published the latest consensus paper on magnetic resonance imaging and radiation exposure in patients with cardiovascular implantable electronic devices [20]. According to the manual of the MicraTM TPS, featured on the official internet side of the company (http://manuals.medtronic.com/manuals/mri/de_AT/search/index), several circumstances are required to perform safe MRI imaging in humans, using either a 1.5 T or a 3 T scanner.

Any abandoned leads, which might still be present from former conventional pacemaker systems, have to be removed. For patients implanted with multiple MR conditional devices, the MR labelling conditions for all implanted devices have to be satisfied. It is required that the SureScan device is operating within the projected service life. Its pacing amplitude has to be ≤4.5 V at the programmed pulse width. Any diaphragmatic stimulation has to be excluded, when “MRI SureScan” is programmed to “On”. If these circumstances are ensured, the device can be switched to “SureScan” mode. Therefore, the operator has to click through a checklist window, which features all parameters of relevance. In patients who require continuous pacing support, the asynchronous pacing mode (VOO) should be selected. Patients who do not require pacing during the MRI examination should be put to the non-pacing mode (OVO). After the MRI examination, the manufacturer recommends a return to the pre-MRI condition as soon as possible, followed by a final check of the pacing capture threshold. If the return is not done within 24 h, the device will end the “SureScan-mode” automatically.

Specific recommendations on static field spatial gradient (<25 T/m), RF exposure (whole body SAR <4 W/kg, Head SAR <3.2 W/kg) and gradient field (gradient slew rate <200 T/m/s per axis) have previously been published by Soejima et al. [21]; however, since the leadless technology is a new therapeutic option, the impact of these devices on the image quality of standard MRI sequences is not yet well known. It is of clinical importance to evaluate whether a diagnostic cardiac MRI would still be feasible after implantation of the Micra system. Therefore, an evaluation of the characteristic appearance of artifacts caused by this leadless pacemaker device is essential. Out of the over 3500 patients with such an implant worldwide, hardly any cases of Micra patients undergoing MRI have been reported. The first published case was a patient with back pain and paralysis, who underwent non-cardiac MRI [22]. To the best of our knowledge only a few isolated cases of cardiac MRI have been reported [23]. Recently, Löbe et al. published a clinical case on cardiac MRI imaging in a patient carrying the other available device [24].

Since a total extraction of the intracardiac device at the end of its lifetime (estimate for Micra approximately 10 years on average) is possible but expected to be challenging, the company propagates the application of a second device. The placement of a second device has already been tested in an animal model by Chen et al. [25] showing effective and safe functionality. Omdahl et al. showed that in human hearts three devices can easily fit even in smaller hearts [26]. We hypothesize that the placement of more intracardiac material might lead to an increase of generated artifacts within cardiac MRI images; however, the huge progression in technology, the rise of the leadless technique and the associated shrinking in size of devices might lead to the development of a second or even third generation system, which might be even smaller. The purpose of our interdisciplinary ex vivo study was to visualize and quantify the generated artifacts and to give an approach on the evaluability of the residual myocardium.

## Material and methods

### Experimental set-up

**Fig. 1:**
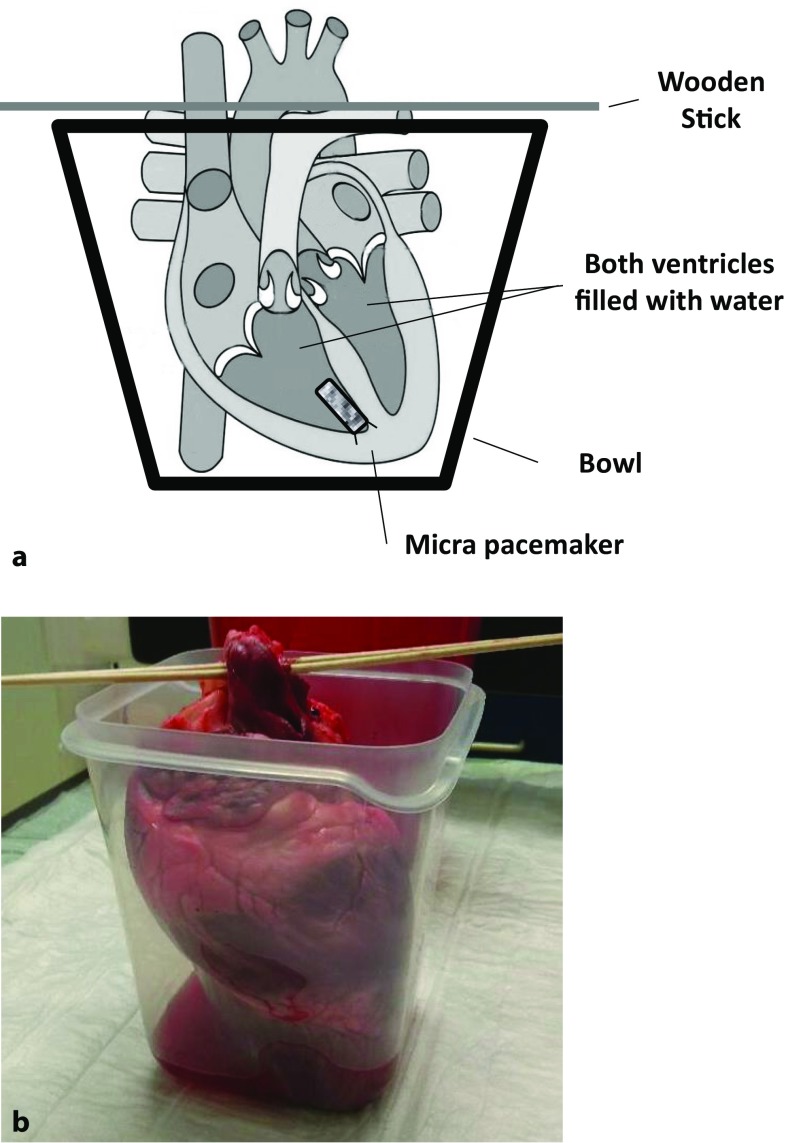
Schematic diagram (**a**) and experimental set-up (**b**) of a porcine heart implanted with the Micra system in the apical region of the right ventricle

A total of 15 domestic pig hearts from a local abattoir were delivered to our laboratory within 2 h after slaughter. A brief visual check for obvious signs of myocardial injury that might have occurred during slaughter was done immediately. The heart as a whole and the great vessels (vena cava, aortic arcus and truncus pulmonalis) were left on bloc. A similar model has already been used to assess the MRI compatibility of temporary pacemaker leads [27]. In a second step, the LP devices were implanted using the original implantation tool via the superior vena cava. The device was maneuvered through the tricuspid valve into the apex of the right ventricle. While applying constant pressure, the pacemaker was deployed through the delivery catheter system and placed in the apicoseptal region of the right ventricle. Hearts were then filled with sodium chloride solution (NaCl 0.9%). To guarantee a stable position and to avoid spilling fluid during the examination, the hearts were placed in bowls and fixed to wooden sticks that were driven through the truncus pulmonalis (see Fig. 1a, b). The space between the heart and the surrounding bowl walls was left free to simulate air filled lungs. Cardiac magnetic resonance (CMR) imaging was performed using a commercially available 1.5 and 3 T scanner (1.5 T Ingenia, 3 T Achieva, Philips Healthcare, Best, Netherlands) and 16 channel anterior-posterior coils were used. Table 1 gives an overview on all used standard sequences.

For visual analysis Agfa Impax EE (R20 XV SU4, Agfa HealthCare GmbH, Bonn, Germany) was used. All images were visually evaluated with respect to whether the LP was displayed in the image for further analysis. The evaluation was performed by a radiologist with experience in cardiac imaging procedures. Computed tomography (CT) scans (Philips 64 CT scanner) and conventional X‑ray studies were conducted.

In total three separate sessions were held using MRI, CT scan and conventional X‑ray, each investigating hearts implanted with the Micra system. All images were selected and interpreted by two radiologists, both experts in cardiac imaging.

This article does not contain any studies with living animals performed by any of the authors.

### Planimetric image analysis

The MR images were imported into image processing software (Adobe Photoshop CS5, Adobe Systems, San Jose, CA, USA). Image J planimetry software (Rasband, W.S., Image J, U.S. National Institutes of Health, Bethesda, MA, USA) was utilized to determine the extent of the artifact area. The size of the artifact area (% of left and right ventricular myocardium) was calculated as follows: the artifact area and the total area of the left and right ventricular area were traced manually in the digital images and measured automatically by the software. Artifact area, expressed as a percentage, was calculated by dividing the area of the artifact by total ventricular area.

## Results

**Fig. 2:**
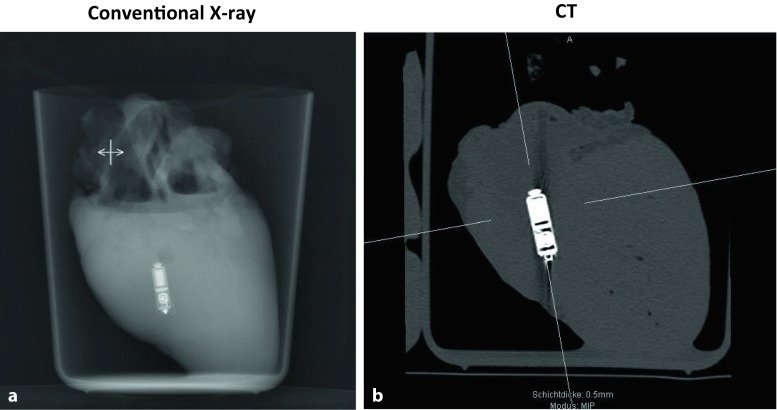
Visualization of the Micra pacemaker system in X‑ray (**a**) and in computed tomography (CT; **b**)

Correct implantation using the steerable device was feasible in all porcine hearts used in our ex vivo study. The experimental set-up of a porcine heart implanted with the Micra system in the apicoseptal region of the right ventricle is shown in Fig. 1a, b. The prepared hearts placed in the open container underwent conventional X‑ray, CT scan and subsequently also MRI in 1.5 and 3 T scanners. Visualization of the Micra pacemaker system in X‑ray is depicted in Fig. 2a and in CT in Fig. 2b.

**Fig. 3:**
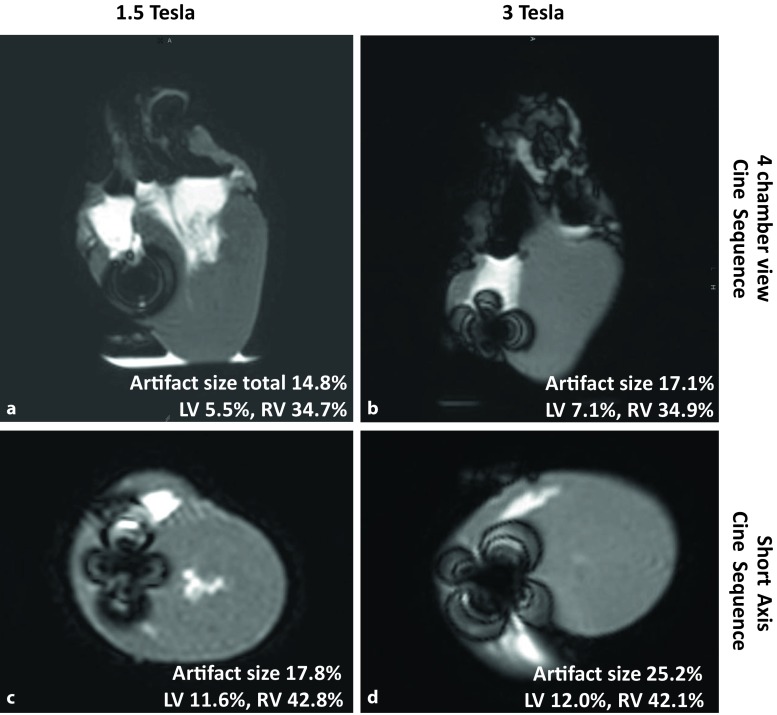
Magnetic resonance imaging in “cine-like” sequences (SSFP-sequence) showing a “shamrock-shaped” artifact masking a small focal area without compromising the surrounding myocardium. The artifact compared to images obtained at 1.5 T (**a** 4-chamber view, **c** short axis view), analysis at 3.0 T showed evidence of a larger visual artifact (**b** 4‑chamber view, **d** short axis view)

Fig. 3 shows MR images in cine-like sequences (steady-state free precession sequence) where the Micra pacemaker system produces a “shamrock-shaped” artifact that masks a small focal area without compromising the surrounding myocardium. The artifact was larger at 3 T (17.1% in long axis and 25.2% in short axis, Fig. 3b, d) than compared to images obtained at 1.5 T (14.8% in long axis and 17.8% in short axis, Fig. 3a, e).

**Fig. 4:**
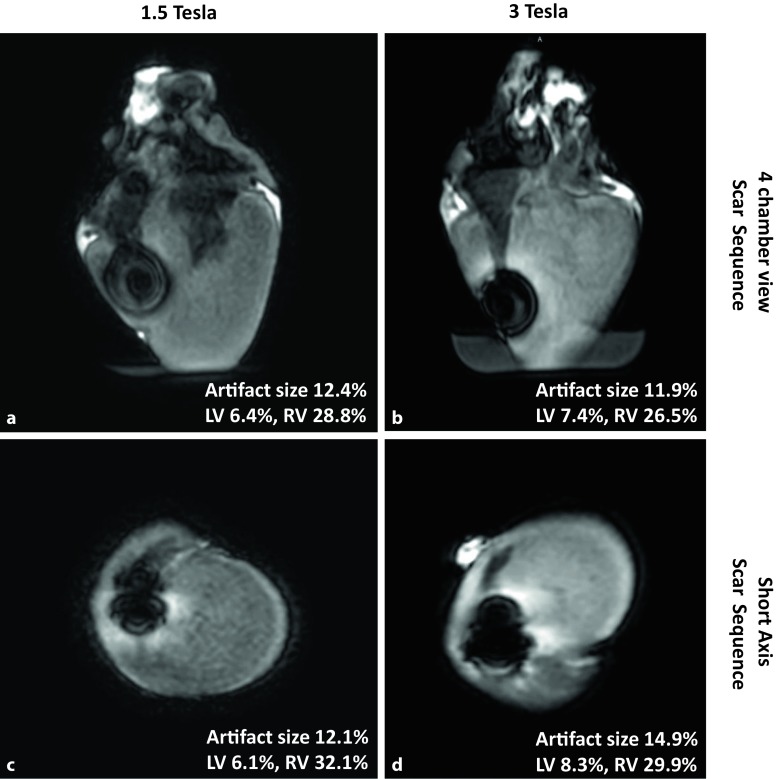
Magnetic resonance imaging scar sequence (**a** scar sequence at 1.5 Tesla, **c** short axis view at 1.5 Tesla) most prone to artifacts at 3.0 T, leaving the right ventricle and the septum affected by a bright, hyperintense perifocal rim (**b** scar sequence at 3 Tesla, **d** short axis view at 3 Tesla)

**Fig. 5:**
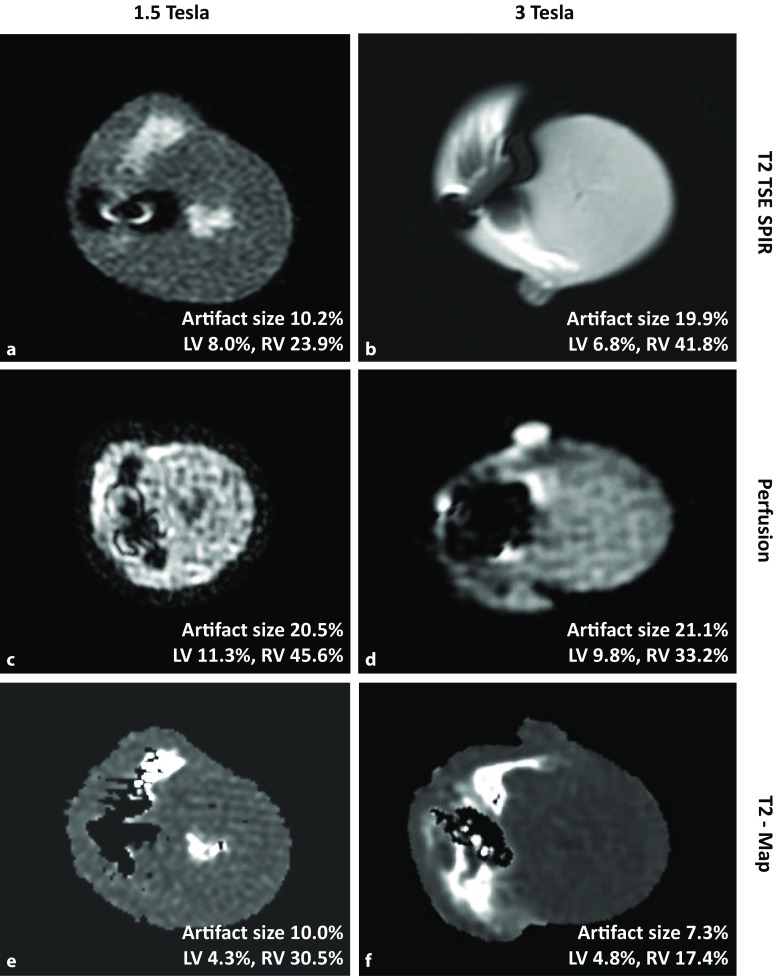
Magnetic resonance imaging T2 TSE-SPIR (**a**, **b**), perfusion (**c**, **d**) and T2 (**e**, **f**) sequences showing few severe artifacts in the perifocal area

A similar result was found for the scar sequence (algorithm for identifying scars in cardiac tissue) (Fig. 4a–d). The late enhancement (scar) sequences were slightly more prone to artifacts at 3.0 T, leaving the right ventricle and the septum affected by a bright, hyperintense perifocal rim (12.4% and 12.1% at 1.5 T vs. 11.9% and 14.9% at 3.0 T; Fig. 4b, d). However, a large proportion of the left ventricular myocardium still remained accessible for image analysis. Few severe artifacts in the perifocal area (Fig. 5) were also visualized in the T2, T2 map and in perfusion sequences at 3.0 T (19.9%, 21.1% and 7.3%, respectively).

Similar findings were generated at 1.5 T. Only the perifocal hyperintense rim artifacts in the turbo-spin-echo (TSE) sequences with selective fat suppression (spectral presaturation with inversion recovery, SPIR) were visually slightly smaller (10.2% vs. 19.9%). No relevant differences were found in perfusion and T2 map sequences.

## Discussion

In recent years, technological advances have set the stage for a completely new era of device treatment. The Micra system was the first pacemaker device with complete intracardiac placement using a femoral access. The purpose of this ex vivo study was to evaluate the suitability of the Micra pacemaker system for MRI scans, as well as to visualize the occurrence of artifacts. Within a small focal area around the device, a shamrock-shaped artifact was generated leading to severely reduced local assessability, while the rest of the myocardium remained suitable for routine radiologic evaluations.

Based on the findings in our ex vivo model we expect good evaluability in major parts of the left ventricle and the lateral wall of the left ventricle, while the septal region and primarily the right ventricle might be overshadowed by focal artifacts. As far as ischemic cardiomyopathy is concerned, diagnosing a notable scar expected in the anterior/anterolateral or lateral myocardium might still be possible without major limitations caused by the device; however, myocardial damage with involvement of the right ventricle and the septum might be poorly assessable and their actual magnitude could eventually be underestimated or overestimated.

In our opinion, diffuse inflammatory cardiomyopathies or myocardial storage diseases affecting large areas of the myocardium, will still be identified; however, the detection and further evaluation of regional myocardial tissue defects will depend on the myocardial location and dimensions.

Pathologies affecting primarily the right ventricle, e.g. arrhythmogenic right ventricular cardiomyopathy (ARVC) might be limited in their assessment by artifacts. Intracardiac masses (thrombus, neoplasms) in the left atrium or in the left ventricle should be possible to visualize and characterize. Since our assumptions are based on observations of a non-vital model, the effects of cardiac motion in real life cannot yet be estimated. Cardiac motion might probably lead to an enlargement of the artifact obscuring the apical region of the right ventricle. We expect no disruptive effects on cardiac valve evaluation.

Compared to sequences at 1.5 T, a remarkable increase in magnitude and intensity of the shamrock-shaped artifact when using a 3 T MRI scanner, could be demonstrated. These effects could especially be seen in the late enhancement (Scar) sequences, which are of extraordinary importance in clinical practice. We would therefore predict that the 1.5 T MRI will be the preferred method of evaluation in clinical settings. In our opinion, limitation to 1.5 T MRI evaluation will not be a major disadvantage in comparison to transvenous systems, as currently conventional systems are usually only approved for 1.5 T.

First clinical data from autopsies showed various forms of intracardiac endothelialization of the device. While some cases showed complete endothelialization within months [28–30], even more unexpected processes of complete encapsulation due to inflammatory processes have been reported [31], while the functionality and the technical parameters of the system remained intact.

The occurrence of ingrowth and encapsulation, might also have an impact on artifact size. Due to the fact that these processes were only seen in single cases so far, the impact on image quality in general will probably remain insignificant. Implantation of a second device, which may become necessary after years of use due to loss of battery function, will obviously lead to an increase of generated artifacts.

Since the magnitude of generated artifacts usually depends more on its metallic components than on its actual size, the effects of a possible new generation device or of an additional LP on MRI remains unclear.

We can presume that in all sequences the right ventricle and the septal region directly surrounding the pacemaker device will show artifacts limiting assessment in a circumscribed area. In these areas, focal signal intensity changes of the myocardium i. e. caused by edema or scar-induced gadolinium (Gd) enhancement might be missed. Focal myocardial thickening or thinning might also be masked. Important to consider is that additional artifacts caused by breathing and cardiac motion will probably further deteriorate the image quality. Considering the estimated battery life of approximately 10 years and the ongoing development of the leadless pacemaker technology, even smaller second generation devices might soon be available. Only time will show the real-life impact of an additional device. Due to the fact that the device did not show any signs of magnetic activity we postulate a safe implementation of cardiac MRI in humans as well. The findings concerning the artefact size might be similar than those in our ex vivo model, when considering the following limitations.

## Limitations

With a total of 15 investigated porcine hearts (*n* = 15, 3 sessions with 5 hearts each), a valuable statistical analysis could not be performed due to the study size being too small. Although the porcine heart is considered to be the most likely model of the human heart, our model obviously has several limitations. Being a non-vital myocardium without any movement, the occurring artifact size might increase when evaluating a vital heart. The impact of cardiac motion on artifacts remains unclear. Even though the experiments were performed immediately after slaughter, a direct comparison to contractile, vital human tissue is not possible. Additionally, minor differences of the myocardial thickness between the human heart and our model have to be considered as well.

In the absence of surrounding lung parenchyma, the normal anatomic setting was simulated by ambient air, which might have an impact on the quality of the images. Furthermore, the hearts were filled with saline, a non-blood-like fluid characterized by a different viscosity. Finally, the fluid remained static within the ventricle, without the flow that would be encountered in the vital setting.

## Conclusion

For the first time, we present a general survey of possible MRI artifacts generated by the intracardiac leadless Micra System. In our ex vivo model, the directly surrounding area of the device showed limited assessability, while the majority of the myocardium remained accessible for routine radiologic MRI evaluations. Since the artifacts appeared to be smaller at 1.5 T than at 3 T in our experimental setting, we suggest a higher diagnostic accuracy at lower field strength. According to this finding we expect that the use of 1.5 T will be the preferred method in clinical practice.

Further clinical studies or even case series would be warranted to estimate the clinical value of MRI in LP patients. From today’s point of view, we postulate that performing a cardiac MRI in patients with a Micra implant is still expedient in selected clinical cases; however, there are limitations concerning the diagnosis of right ventricular pathologies.

**Table 1:** MRI p CMR protocol. CMR imaging was performed using a commercially available 1.5 and 3 T scanner (1.5 T Ingenia, 3 T Achieva, Philips Healthcare, Best, Netherlands)

	1.5 T (Ingenia, Philips Healthcare, Best, Netherlands)	3 T (Achieva, Philips Healthcare, Best, Netherlands)
“Cine-like” images (in static heart) 4CH	Repetition time (TR) = 3.39 ms	Repetition time (TR) = 2.16 ms
	Echo time (TE) = 1.7 ms	Echo time (TE) = 1.08 ms
	Flip angle (FA) = 60°	Flip angle (FA) = 45°
	FOV = 350 × 350 mm^2^	FOV = 320 × 348 mm^2^
	Matrix = 208 × 198	Matrix = 180 × 197
	Slice thickness = 8 mm	Slice thickness = 8 mm
“Cine-like” images (in static heart) SAX	Repetition time (TR) = 3.04 ms	Repetition time (TR) = 2.08 ms
	Echo time (TE) = 1.52 ms	Echo time (TE) = 1.04 ms
	Flip angle (FA) = 60°	Flip angle (FA) = 45°
	FOV = 350 × 350 mm^2^	FOV = 320 × 348 mm^2^
	Matrix = 176 × 171	Matrix = 180 × 210
	Slice thickness = 8 mm	Slice thickness = 8 mm
T2 TSE SPIR SAX	Repetition time (TR) = 211.1 ms	Repetition time (TR) = 2.08 ms
	Echo time (TE) = 60 ms	Echo time (TE) = 1.04 ms
	Flip angle (FA) = 90°	Flip angle (FA) = 45°
	FOV = 350 × 350 mm^2^	FOV = 320 × 348 mm^2^
	Matrix = 232 × 155	Matrix = 180 × 210
	Slice thickness = 8 mm	Slice thickness = 8 mm
Perfusion SAX	Repetition time (TR) = 2.3 ms	Repetition time (TR) = 2.18 ms
	Echo time (TE) = 1.14 ms	Echo time (TE) = 0.7 ms
	Flip angle (FA) = 50°	Flip angle (FA) = 18°
	FOV = 360 × 360 mm^2^	FOV = 380 × 368 mm^2^
	Matrix = 128 × 120	Matrix = 128 × 124
	Slice thickness = 8 mm	Slice thickness = 8 mm
SCAR/LE Sequence 4CH	Repetition time (TR) = 3.24 ms	Repetition time (TR) = 3.37 ms
	Echo time (TE) = 1.58 ms	Echo time (TE) = 1.68 ms
	Flip angle (FA) = 15°	Flip angle (FA) = 15°
	FOV = 340 × 303 mm^2^	FOV = 390 × 335 mm^2^
	Matrix = 220 × 186	Matrix = 256 × 195
	Slice thickness = 10 mm	Slice thickness = 10 mm
SCAR/LE Sequence SAX	Repetition time (TR) = 3.45 ms	Repetition time (TR) = 3.27 ms
	Echo time (TE) = 1.67 ms	Echo time (TE) = 1.64 ms
	Flip angle (FA) = 15°	Flip angle (FA) = 15°
	FOV = 390 × 311 mm^2^	FOV = 390 × 335 mm^2^
	Matrix = 256 × 182	Matrix = 256 × 195
	Slice thickness = 10 mm	Slice thickness = 10 mm

*4CH* 4 chamber view, *FOV* field of view, *LE* late enhancement, *SAX* short axis view, *SCAR* car sequence, *T2-TSE-SPIR SAX* turbo spin echo-spectral preseturarion with inversion recovery
